# The Impact of T Cell Intrinsic Antigen Adaptation on Peripheral Immune Tolerance

**DOI:** 10.1371/journal.pbio.0040340

**Published:** 2006-10-17

**Authors:** Nevil J Singh, Chuan Chen, Ronald H Schwartz

**Affiliations:** Laboratory of Cellular and Molecular Immunology, National Institute of Allergy and Infectious Diseases, National Institutes of Health, Bethesda, Maryland, United States of America; National Jewish Medical and Research Center, United States of America

## Abstract

Overlapping roles have been ascribed for T cell anergy, clonal deletion, and regulation in the maintenance of peripheral immunological tolerance. A measurement of the individual and additive impacts of each of these processes on systemic tolerance is often lacking. In this report we have used adoptive transfer strategies to tease out the unique contribution of T cell intrinsic receptor calibration (adaptation) in the maintenance of tolerance to a systemic self-antigen. Adoptively transferred naïve T cells stably calibrated their responsiveness to a persistent self-antigen in both lymphopenic and T cell–replete hosts. In the former, this state was not accompanied by deletion or suppression, allowing us to examine the unique contribution of adaptation to systemic tolerance. Surprisingly, adapting T cells could chronically help antigen-expressing B cells, leading to polyclonal hypergammaglobulinemia and pathology, in the form of mild arthritis. The helper activity mediated by CD40L and cytokines was evident even if the B cells were introduced after extended adaptation of the T cells. In contrast, in the T cell–replete host, neither arthritis nor autoantibodies were induced. The containment of systemic pathology required host T cell–mediated extrinsic regulatory mechanisms to synergize with the cell intrinsic adaptation process. These extrinsic mechanisms prevented the effector differentiation of the autoreactive T cells and reduced their precursor frequency, in vivo.

## Abstract 2

Intrinsic adaptive mechanisms can keep T cells from responding to chronic antigenic stimulation (thus reducing the chances of autoimmunity), but cell-extrinsic mechanisms (such as clonal deletion) are needed to enforce true tolerance.

## Introduction

The efficient clonal expansion of pathogen-specific T cells plays a crucial role in determining the success of an immune response against a rapidly replicating infectious challenge. The ability of this expanded lymphocyte pool to effectively combat the pathogen also relies on the extent of effector functions that it acquires and maintains. Differentiated helper T cells produce cytokines and cell surface ligands that regulate the subsequent generation of cytotoxic and humoral responses. This differentiation process is often correlated with proliferative expansion, but there is evidence to suggest that the two processes can be independently regulated [1–3]. Subsequent to clearance of the pathogen, most members of these expanded populations of antigen-specific lymphocytes are eliminated and the few that survive often typically demonstrate better responsiveness.

In cases where a T cell response is initiated against a chronic nonclearable pathogen or a persistent self-antigen, the immune system evokes many regulatory mechanisms aimed at containing the potentially damaging chronic T cell activity. One such mechanism has been called adaptive tolerance [4]. This process is a T cell–intrinsic downregulation of responsiveness, likely mediated by the recruitment of negative feedback in signaling pathways downstream of the T cell receptor (TCR). The consequent hyporesponsiveness of the T cell is proportional to the strength of the ambient antigen presentation and is reversible upon removal from the antigen-bearing host [5–7]. Such a dynamic state is broadly consistent with the tunable activation threshold model originally proposed by Grossmann and Paul [8] and may allow for the persistence of autoreactive T cells that are potentially useful against foreign antigens [9]. We have earlier shown that this antigen adaptation primarily aims to restrict the turnover of T cells in vivo to a minimal basal level, despite the persistence of antigen [6]. The T cells that enter the hyporesponsive state, however, have undergone significant differentiation and can produce effector cytokines at levels higher than naïve T cells (albeit lower than memory T cells) after an in vitro restimulation. This raises the possibility that antigen-adapted T cells may continue to chronically display effector functions against the persistent antigen despite the restriction of their proliferative ability.

The downregulation of the proliferative potential of helper T cells, while maintaining their ability to mediate effector functions, has been reported in the case of T cells surviving an acute antigen exposure in the absence of adjuvant [10]. In this model, the tolerizing antigen does not persist and therefore the effector potential of the T cells is unlikely to be stimulated to induce pathology. It is therefore not clear if continued persistence of antigen would result in the elimination of the T helper cell's effector function as well. Furthermore, CD8^+^ T cells that undergo adaptation to chronic lymphocytic choriomeningitis virus (LCMV) infection or a self-antigen downregulate both their proliferative and effector functionalities [11,12]. In this case, the ability to produce interleukin (IL)-2 was often downregulated rapidly, while various effector functions required extended stimulation through chronic viral exposure [13]. CD8^+^ T cells experiencing chronic antigen in a transgenic model, however, retained the ability to mediate cytolytic activity in vivo despite anergy induction [14]. In the early phases of a chronic LCMV infection, CD4^+^ T cells specific for the virus were able to help antigen-expressing (infected) B cells polyclonally, leading to serum hypergammaglobulinemia [15]. This antibody production correlates with the acute viremia and suggests that after several weeks of chronic viral infection, CD4^+^ T cells lose the ability to help B cells [16]. Nonetheless, the fluctuations in the antigen load due to viral replication, clearance and tissue redistribution also makes such models less suited to examination of the in vivo functionality of stably antigen-adapted T cells.

We have previously described a model system for adaptive tolerance that uses transgenic mice constitutively expressing the antigen pigeon cytochrome C (PCC), driven by the MHC class I promoter and an Ig enhancer, in a variety of cellular compartments, including B cells and dendritic cells [6,7,17]. Antigen-specific naïve CD4^+^ T cells adoptively transferred into such mice crossed onto a CD3ɛ^−/−^ background, adapt to the persistent antigen over the period of a week. In the absence of other competing T cells, these CD4^+^ cells persist for extended periods of time in the adaptively tolerant state and show no evidence of having acquired a T-reg phenotype [6,7]. This system, therefore, allows us to examine the consequence of antigen adaptation on the T cell's ability to mediate effector functions in response to the ambient stimulation, for example, by helping B cells that express and present the cytochrome C antigen.

In this report, we demonstrate that the CD4^+^ T cell expansion to the persistent self-antigen results in a polyclonal antibody response by the resident B cells. The antibodies are directed against a variety of self-antigens classically implicated in autoimmune disorders. The mice eventually develop a mild form of chronic arthritis. Interestingly, CD4^+^ T cells that were first stably adapted to the antigen in the absence of B cells still retained the ability to help B cells and to cause autoimmune disease. In contrast, in the presence of an endogenous repertoire of T cells, the ability of the autoreactive T cell to induce pathology was severely compromised. This tolerance was correlated with a failure of the transferred transgenic T cells to complete effector differentiation and survive in the T cell–replete host. Overall, these data demonstrate that at least two distinct mechanisms—one cell intrinsic and the other in trans—must cooperate to ensure complete peripheral tolerance.

## Results

### Adaptation Controls Autoreactivity Independent of Suppression

The exposure of naïve helper T cells to chronic peripheral antigenic stimulation leads to a reversible state of anergy known as adaptive tolerance. We have previously modeled this process by adoptively transferring Rag2-deficient 5C.C7 TCR transgenic T cells into CD3ɛ knockout mice that express membrane-targeted PCC under the control of an H-2K promoter and an Ig enhancer [17]. After a robust initial expansion, the transferred T cells downregulate their ability to make cytokines to an in vitro restimulation and fail to clonally expand well upon retransfer into a fresh mPCC,CD3ɛ^−/−^ host [6,7].

**Figure 1:**
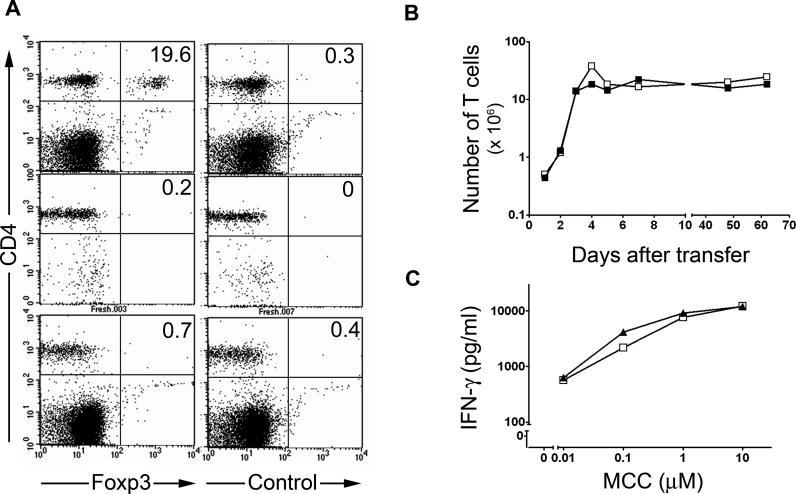
Adaptive Tolerance Can Be Induced Independently of Regulatory Activity (A) Intracellular staining with antibodies to *foxp3* (left panels) or an isotype-matched control (right panels) on naive 5C.C7 T cells (middle two panels) or antigen-adapted T cells (bottom two panels). Positive control staining in a polyclonal population of B10.A T cells marks approximately 19% of the CD4^+^ T cells (top two panels). (B) A new cohort of naive 5C.C7 T cells expands similarly in B10.A, PCC^+/+^, CD3ɛ^−/−^ hosts (open squares) even if they already harbor expanded 5C.C7 T cells (solid squares). Newly transferred 5C.C7 T cells were distinguished from the previous population using congenic markers (Ly5.1). (C) Congenically marked Ly5.1^+/+^,5C.C7 T cells that expanded in a naive B10.A, PCC^+/+^, CD3ɛ^−/−^ host (open squares) or a PCC^+/+^, CD3ɛ^−/−^ host that had been injected with Ly5.2 ^+/+^,5C.C7 T cells 28 d in advance (solid squares) were purified by FACS sorting (Ly5.1+, CD4+) and restimulated in vitro to measure IFNγ secretion.

Proliferative impairment in peripheral T cells after autoantigen encounter has often been attributed to the induction of a regulatory phenotype in T cells [18,19]. Since the host mice in our experiments do not have any endogenous T cells, by design (CD3ɛ^−/−^), we examined if the adaptively tolerant 5C.C7 T cells acquired a regulatory function in the host. As shown in Figure 1A, we could not detect *foxp3* expression in the transferred cells at any time that we tested (days 4, 11, 22, and 28, of which day 11 is shown in Figure 1A, bottom row). Regulatory activity may still act through a *foxp3* independent fashion, but would be expected to either impair the proliferation or differentiation of a new T cell response [20]. We tested this by adoptively transferring fresh Ly5.1^+^ naïve 5C.C7 T cells into mPCC,CD3ɛ^−/−^ mice that already harbored Ly5.2^+^ 5C.C7 T cells in an adaptively tolerant state. The resident population was unable to impair the first 3 d of expansion of the fresh naïve cohort (Figure 1B, days 1–3). After this time, the new cohort did not expand significantly and could be recovered at similar numbers to that in a lymphopenic host (solid squares in Figure 1B). Finally, in order to examine if the differentiation of the second cohort was impaired by the presence of adaptively tolerant 5C.C7 T cells, we purified the expanded second cohort (Ly5.1^+^, 5C.C7) by FACS sorting. The sorted cells were assayed for their ability to produce interferon (IFN)γ after in vitro restimulation. Ly5.1^+^ 5C.C7 T cells that expanded in the presence of adapted Ly5.2^+^ 5C.C7 T cells (solid triangles in Figure 1C) or in an empty mPCC,CD3ɛ^−/−^ host (open squares in Figure 1C) had differentiated similarly as evidenced by effector cytokine production in vitro. These data suggest by multiple criteria, that adaptive tolerance in this model, is a cellular phenomenon distinct from suppression. It is also independent of clonal deletion, since the adaptively tolerant T cells are not eliminated in the mPCC,CD3ɛ^−/−^ host for the life of the animal—solid squares in Figure 1B. Therefore, our model system allows for a direct evaluation of the impact of antigen adaptation on systemic immunological tolerance, unlike many other models in which multiple paradigms cannot be resolved sufficiently.

### Adaptation Alone Is Insufficient to Generate Systemic Tolerance

**Figure 2:**
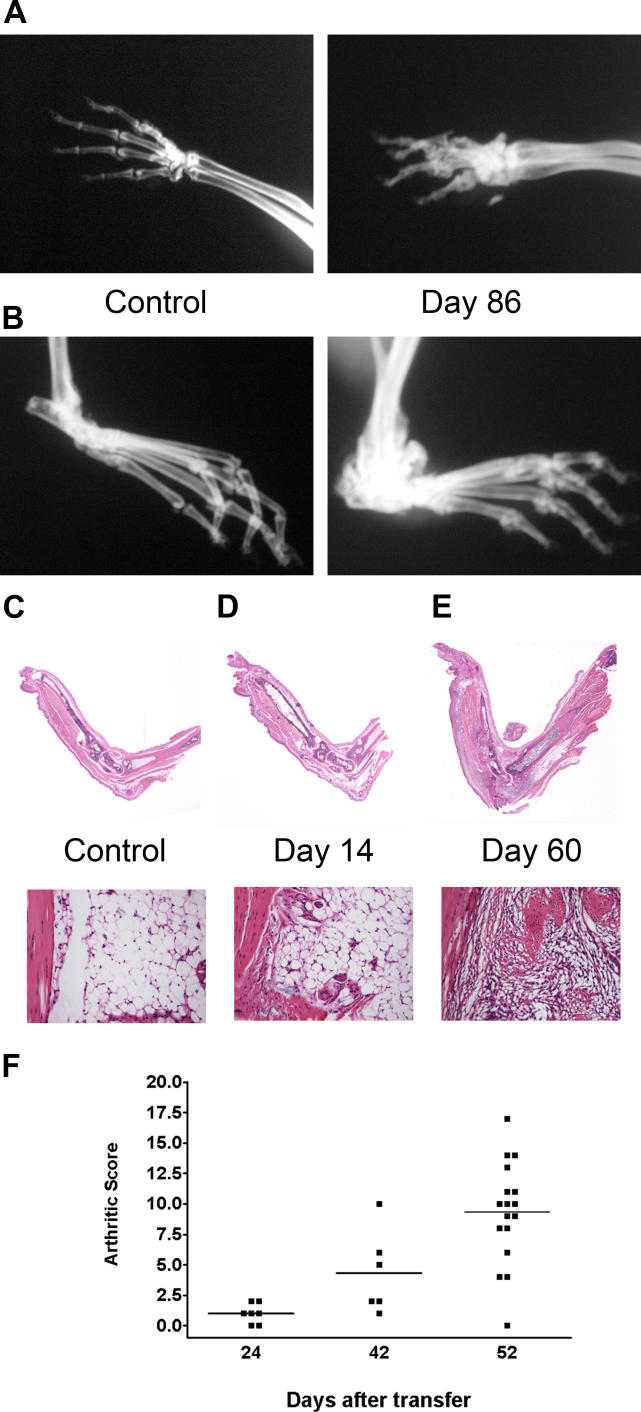
Adoptively Transferred T Cells Induce Arthritic Pathology in Recipient Mice (A) X-ray radiographs of fore limbs and (B) and hind limbs comparing mPCC,CD3ɛ^ −/−^ mice without a T cell transfer (left panels) and one 86 d after (right panels). (C–E) Hindlimbs from mPCC,CD3ɛ^ −/−^ mice were sectioned and stained by HEMATOXYLIN AND EOSIN before (C) T cell transfer and 14 d (D) or 60 d (E) afterward. Lower panels show magnification of the joint regions from the top panels, to illustrate cellular infiltrates in the tibiotarsal region. (F) Clinical scores quantitate increased limb pathology in transfer recipients on a scale of 0 to 56. Bars represent arithmetic means in each group.

The mPCC,CD3ɛ^−/−^ mice that harbor the tolerant PCC-reactive 5C.C7 T cells do not display any obvious signs of acute graft versus host disease such as weight loss, lethargy, paralysis, or lethality. Interestingly, however, we began to observe swelling of the hindlimbs and forelimbs of a few mice, almost 2 mo after the adoptive transfer. These phenotypes were observed only in the PCC transgenic recipients and not in control CD3ɛ^−/−^ mice, despite a weak “homeostatic” expansion of the 5C.C7 T cells, reported earlier [6]. To further characterize this phenotype, we examined the animals by x-ray imaging (Figure 2A and 2B) and Immunohistochemistry (Figure 2C–2E). Radiographs of the forelimb of an adoptive transfer recipient at day 86 showed bone loss with significant distortion of the phalanges (Figure 2A, right) relative to a control mouse (Figure 2A, left). The hindlimb (Figure 2B) also showed increased bone density especially around the carpal joints. Hindlimbs of mPCC,CD3ɛ^−/−^ mice that were infused with autoreactive T cells 14 d earlier (Figure 2D) or 60 d earlier (Figure 2E) were stained by HEMATOXYLIN AND EOSIN after decalcification and sectioning. The sections show the increased swelling at 60 d (Figure 2E) compared to both the control (Figure 2C) and the 14-d time point (Figure 2D). The legs were extended maximally before being processed for sections and the lack of extension in the day 60 leg is also evident in Figure 2E. Furthermore, mild mononuclear cell infiltrates in the tibiotarsal regions were evident in magnifications of the day 60 sections (Figure 2E, bottom). These findings suggested that an arthritic process was developing in the 5C.C7 transfer recipients, despite the induction of adaptive tolerance in these T cells. This was surprising since the B10.A strain is not related to genotypes that are conventionally associated with the ability to develop arthritis, even after collagen immunization [21]. Although the severity of the pathology did not approach that of some arthritic models [22,23] or significantly hamper feeding or movement of the mice, we developed a scoring system to quantitate this swelling (described in Materials and Methods). Subtle alterations in joints or digits were visible in a few mice as early as 24 d after transfer and progressively increased over time (Figure 2F). Although not completely penetrant, up to 80% of the transfer recipients developed disease scores over 5 in less than 2 mo.

### Polyclonal Activation of B Cells Drives an Autoreactive Response during Adaptive Tolerance Induction

**Figure 3:**
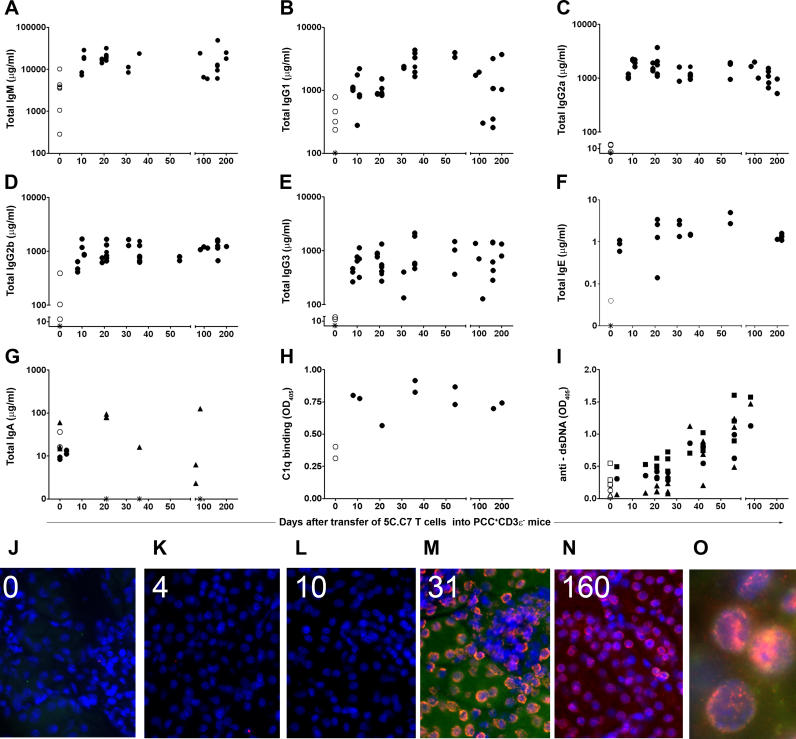
Polyclonal B-Cell Activation and Autoantibody Production following the Induction of T Cell Adaptive Tolerance (A–G) Total serum antibody levels of individual isotypes, measured by sandwich ELISA at different days after T cell transfer (solid circles). Open circles at time 0 represent serum measurements from normal mice, without T cell transfer. *Serum samples where the levels of the isotype was below detection [for two, four, three, three, and six samples each in (B), (C), (D), (E), and (F)]. Data in (A–E) are combined from two experiments between 4 and 60 d and two additional experiments at later time points, with two to five mice per experimental group. Three additional experiments between days 7 and 120 gave similar results. Data in (F) and (G) are from two separate experiments with three mice per time point. Serum IgA levels were below our limit of detection after the day 3 time point as indicated (*). Solid triangles in (G) represent measurements from solubilized fecal pellets. Note that the *y*-axes are scaled differently for (A), (F), and (G) to accommodate different absolute amounts of IgM, IgE, and IgA. (H) Increased circulating immune complexes in the serum after T cell transfer, demonstrated by a C1q binding ELISA. Each data point represents an individual mouse, whose sera were all sampled on the same day after receiving T cells at various prior times (*x*-axis). (I) Titers of anti-DNA antibodies in the sera of 16 individual T cell transfer recipients sampled in two separate experiments. Optical density values obtained from isotype specific ELISAs for IgM (solid circles), IgG2b (solid squares), and IgG2a (solid triangles) are shown. Empty circles (IgM), squares (IgG2b), and triangles (IgG2a) on day 0 represent sera obtained from three normal mice, assayed in the two experiments. (J–O) Sera from mPCC,CD3ɛ^−/−^ mice were assayed for anticellular antibodies of the IgM (green) and IgG (red) isotypes, by hybridizing with frozen tester sections of B10.A,Rag2^−/−^ kidneys. Numbers in the inset denote days after T cell transfer. (O) shows an enlarged area from (N) to illustrate speckled nuclear staining. (J) was probed with serum from a normal mPCC,CD3ɛ^−/−^ mouse without T cell transfer. Similar sera samples from mice 4 d (*n* = 3) or 7 d (*n* = 2) after T cell transfer did not show measurable IgG or IgM anti cellular antibodies. However, all mice tested after 28 d (*n* = 22) scored positive for both IgG and IgM antibodies to various extents.

T cell–initiated autoimmune syndromes in mouse models often require B-cell–mediated antibody production or antigen presentation [24]. We examined whether this was the case in our model system, by assaying serum antibody levels in PCC transgenic mice at various days after the transfer of 5C.C7 T cells (Figure 3). The total amount of IgM in the serum had increased to approximately 5-fold by 21 d of 5C.C7 T cell transfer (Figure 3A, *p* < 0.0001 comparing normal serum values at day 0 with day 21 after T cell transfer). This increased level was maintained stably up to 200 d after the initial transfer. A similar pattern was observed for total amounts of IgG1 (Figure 3B), IgG2a (Figure 3C), IgG2b (Figure 3D), IgG3 (Figure 3E), and IgE (Figure 3F). All increases were statistically significant over the levels detected in control mice (on day 21, *p* = 0.0024 for IgG1, *p* < 0.001 for IgG2a, *p* < 0.001 for IgG2b, and *p* = 0.01 for IgG3). The increase in these isotypes, however, was more dramatic, because little or no production was detected prior to the T cell infusion [open circles in (A)–(E), * indicates samples with undetectable levels]. The increase was also antigen specific since we did not see a comparable increase in Ig levels after the transfer of 5C.C7 T cells to B10.A,CD3ɛ^−/−^ mice that did not express PCC (unpublished data). The global increase in the amounts of all of these isotypes shows that there is no bias toward any “class” of immune response. This is also consistent with our earlier report of a global suppression of cytokine production in the adaptively tolerant T cells, without skewing toward any particular subset or class of response. The levels of IgA, however, were distinct from those of all the other isotypes (Figure 3G). There was no increase in the fecal levels of IgA (triangles) after T cell transfer, and there was a reproducible decrease in the basal IgA levels detected in the serum (solid circles). Consistent with increased antibody production in these mice, we could also detect an increased amount of circulating antigen-antibody complexes as measured by a complement (C1q) binding ELISA (Figure 3H).

We also examined the reactivity of antibodies generated in this system against a panel of mouse antigens. Antibodies could be detected against double-strand DNA (Figure 3I) as well as ribosomal antigen extracts (unpublished data). The levels of anti-DNA antibodies in the serum of mice 56 d after 5C.C7 transfer were significantly enhanced over control mice in all three isotypes tested: IgM (*p* = 0.01), IgG2a (*p* = 0.02), and IgG2b (*p* = 0.02). Sera from transfer recipients were tested on Western blots for reactivity against purified antigens. Although the private reactivities varied between individual mice (Table 1), most animals produced detectable antibodies against multiple antigens, including histones, glucose-6-phosphate isomerase (GPI), fibrinogen, and collagen. Occasionally, antibodies could also be detected against PCC, consistent with the idea that B cells in this transgenic are not completely tolerant of PCC, in the presence of T cell help [25].

**Table 1:**
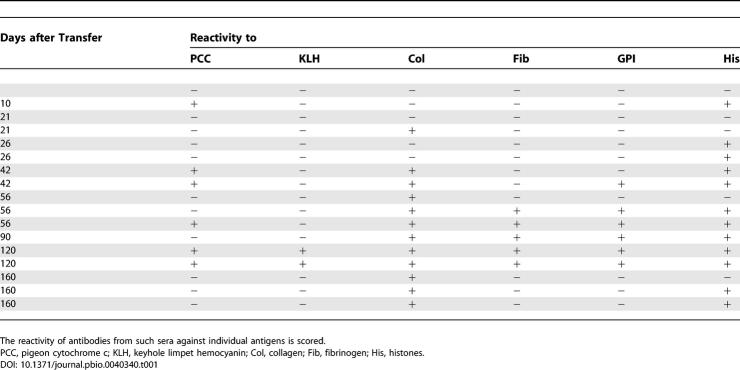
Purified Antigens Separated by SDS-PAGE and Transferred to Nitrocellulose Membranes Were Probed with Serum Samples (1:1,000 Dilution) from Mice Bled Various Days after T cell Transfer

This broad spectrum of anticellular antibodies was also evident in immunofluorescence assays on test sections of healthy murine kidneys (Figure 3J–3N). Sera obtained 4 wk after transfer consistently (*n* = 22 mice) showed reactivity against cellular antigens. The staining showed a speckled subnuclear reactivity (shown by higher magnification in Figure 3O) that is consistent with patterns reported from mice demonstrating autoimmune B-cell pathology, including SLE. It is also noteworthy that although the total Ig levels were almost maximally induced by day 10 (Figure 3A–3F), there were no detectable anticellular antibodies until after 4 wk (Figure 3M). Thus, there was a qualitative change in the antibodies being made at later time points, even after the amount of secreted antibody had peaked (Figure 3K–3N shows progressive changes in reactivity).

### The Help for B Cells Is Available Even after the Stable Induction of Adaptive Tolerance

**Figure 4:**
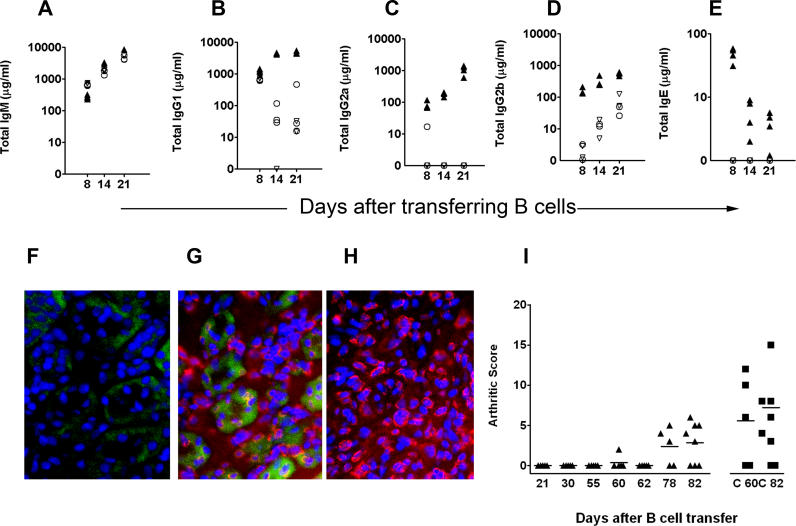
Stable Adaptive Tolerance Does Not Abrogate the Ability of T Cells to Help Autoreactive B Cells (A–E) Purified splenic B cells (25 million) were transferred into mPCC,Rag2^−/−^ mice that received 5C.C7 T cells 48 d earlier. The amount of serum antibody of each isotype 8, 14, and 21 d after the transfer of PCC transgenic B cells (triangles) or antigen-free B cells (circles) is plotted. Solid triangles and open circles are measurements from mice that received 5C.C7 T cells 48 d before the B-cell transfer. Open inverted triangles are measurements from mPCC,Rag2^−/−^ mice that did not receive T cells. Two other experiments, including one using FACS-sorted B cells, showed similar results. (F–H) Antinuclear antibodies in the serum of mPCC,Rag2^−/−^ mice 30 d after B-cell transfer (H). Three other mice from the same experiment also showed anticellular reactivity. Similarly stained tester sections using serum from an mPCC,CD3ɛ^ −/−^ mouse that received 5C.C7 T cells 60 d earlier (G) or a control B10.A mouse (F) are provided for comparison. (I) Arthritis development between 21 and 82 d after the transfer of mPCC^+^ B cells (triangles) into mPCC,Rag2^−/−^ mice bearing adoptively tolerant 5C.C7 T cells. Arthritis in mPCC,CD3ɛ^−/−^ mice 60 and 82 d after receiving naive 5C.C7 T cells (squares: C 60, C 82) are scored for comparison.

The antibody responses observed in the 5C.C7 transfer recipients could have been triggered by the robust, early (days 1–4) expansion of naïve T cells in the mPCC,CD3ɛ^−/−^ host (Figure 1B, open squares), because stable adaptation to antigen (as measured by cytokine downregulation) takes approximately 1 wk to establish in this system [26]. We therefore devised an experimental strategy to test if T cells could in fact mediate B cell help even after the stable induction of adaptive tolerance. We took advantage of the fact that naïve 5C.C7 T cells can be induced into a stable adaptively tolerant state in the absence of B cells, by using an mPCC,Rag2^−/−^ host for the adoptive transfers. (N. J. Singh, L. Chiodetti, and R. H. Schwartz, unpublished results). We waited 48 d after transfer of T cells into such hosts and then transferred 25 million naïve B cells intravenously (Figure 4). B cells were purified from the spleens of mPCC,CD3ɛ^−/−^ mice (that express the antigen) or control CD3ɛ^−/−^ mice (that do not express PCC). Transfer recipients were bled at various days after B-cell transfer and serum immunoglobulins measured in an isotype-specific manner (Figure 4A–4E).

Serum IgM levels in the transfer recipients increased steadily after B-cell transfer, and there was only a subtle increase afforded by antigen expression in the transferred B cells (Figure 4A, solid triangles). In contrast, there were significant antigen-specific increases in IgG1 (*p* < 0.001 on day 14) and IgG2a levels (*p* < 0.001 on day 14) only observed in the presence of adapted T cells (Figure 4B and 4C). In the case of IgG2b, although there was a 5- to 10-fold increase in antibody production with PCC-expressing T cells (Figure 4D, solid triangles), the transfer of B cells alone resulted in increased total IgG2b levels in the absence of any T cells (Figure 4D, open inverted triangles). However the antigen-specific increase was still significantly greater (*p* = 0.05 on day 14, Figure 4D, closed triangles). In the presence of adapted T cells and antigen-expressing B cells, there was a sharp increase in IgE levels as early as day 8 and a consistently increased but lower level thereafter (Figure 4E, solid triangles). We could not detect IgE production in the recipients of non–antigen-expressing B cells or in the absence of adapted T cells (Figure 4E, open circles and triangles). These data demonstrate that even 48 d after T cell transfer into a self-antigen–expressing environment, adaptively tolerant T cells retain the ability to robustly help B cells in an antigen-specific fashion.

The increase in serum immunoglobulin levels in the T cell reconstituted B10.A,mPCC,CD3ɛ^−/−^ mice precedes the development of both anticellular antibodies (Figure 3) and visible arthritis (Figure 2). Therefore, it was still possible that the adapted T cells would be unable to aid the complete transition from increased serum titers to developing joint disease. We examined this possibility by introducing PCC transgenic B cells into mPCC,Rag2^−/−^ mice, 28 d after they had received a 5C.C7 T cell transfer. We then assayed serum anticellular antibodies and arthritis in the recipients at various days. The serum anticellular antibody levels in these mice were similar to those of B10.A,mPCC,CD3ɛ^−/−^ mice (compare Figure 4G to Figure 4H). However, the arthritis (Figure 4I, solid triangles) was clearly delayed, since no significant joint swelling or rigidity was observed up to 60 d after transferring B cells (88 d after the T cell input). These mice did start to develop measurable disease by 80+ d; however, the severity was less than that of B10.A,mPCC,CD3ɛ^−/−^ mice at 60 d after T cell reconstitution (Figure 4I, solid squares). These observations demonstrate that the antigen-adapted T cell population can still facilitate the maturation of the B-cell response that leads to the production of anti–self-reactive antibodies.

### Adaptively Tolerant T Cells Mediate Help through Cytokines and CD40–CD40L Interactions

T cell help to B cells is mechanistically delivered through the secretion of cytokines (including IL-4, IL-5, IL-21, IFNγ, etc.) and the engagement of cell surface receptors (including CD40). Since adaptation dampens TCR signaling in these T cells [27], the expression of these molecules may also be expected to be impaired. However, T cells have undergone significant differentiation to make effector cytokines by the time they are rendered tolerant [6]. The help that we observe could therefore result from either the low levels of effector molecules that are made despite adaptive tolerance induction or alternatively by the unaffected synthesis of any one component, which is resistant to the dampening effects of adaptive tolerance. We tested these two possibilities by knockout and blocking strategies designed to ask if any one component was the dominant inducer of B-cell differentiation in this model system.

**Figure 5:**
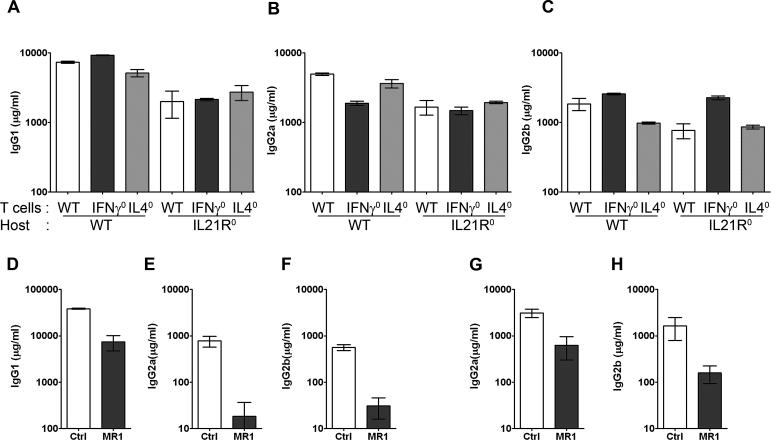
Cytokine and CD40L Dependence of Help Rendered by Adaptively Tolerant T Cells (A–C) Serum from mice that received T cells 8 d before were assayed for total immunoglobulins as described. T cells were from 5C.C7 TCR transgenic mice (white bars) or similar mice also deficient in IFN-γ (black bars) or IL-4 (gray bars). Host mice were mPCC,CD3ɛ^−/−^ (left three bars in each graph) or IL-21 receptor–deficient mPCC,CD3ɛ^−/−^ mice (right three bars). (D–F) B cells were infused into mPCC,Rag2^−/−^ mice that harbored stably adapted 5C.C7 T cells. The blocking anti-CD40L antibody MR1 (black bars) or a control hamster antibody (white bars) was administered at the same time and on every other subsequent day for 13 d, before assaying serum for antibody titers. (G, H) Similar experiments (as in D–F) were carried out, but the blocking antibody treatment was terminated at 6 d. Twenty-two days afterward, serum IgG2a (G) and IgG2b (H) levels were measured in the MR1 treated (black bars) or control (white bars) mice.

The absence of IL-4 in the transferred 5C.C7 T cells (Figure 5A–5C, gray bars) resulted in less than 2-fold decrease in serum IgG1 (*p* = 0.08, in Figure 5A) and IgG2b (*p* = 0.05, Figure 5C) levels. Similarly, IFNγ knockout 5C.C7 T cells elicited approximately 2- to 3-fold lower levels of IgG2a (*p* = 0.006, Figure 5B) with a slight increase in IgG1 (*p* = 0.02). The cytokine IL-21 has been reported to act as a Th2 enhancer or plasma cell factor in differentiating B cells [28,29]. We used mPCC,CD3ɛ^−/−^ mice that were also deficient in IL-21 receptor as transfer recipients to examine the contribution of this cytokine to the hypergammaglobulinemia. The absence of IL-21 responsiveness in B cells decreased serum levels of all isotypes by 2- to 3-fold (Figure 5A–5C, last three bars in each graph). Interestingly combining IL21R deficiency in B cells together with IL-4 or IFNγ deficiency in T cells did not synergistically reduce serum Ig levels any further.

We then examined the role of CD40–CD40L interactions using the mPCC,Rag2^−/−^ model described in the previous section. Mice with resident adaptively tolerant T cells were treated with blocking anti-CD40L antibody (MR1) or a control antibody intraperitoneally (100 μg/mouse) at the same time as introducing naïve PCC-expressing B cells (Figure 5D–5F). The blocking antibody treatments were continued every other day and the serum antibody titers measured 14 d after B-cell transfer. In contrast to the cytokine deficiency experiments, blocking CD40–CD40L interactions resulted in a profound decrease in IgG2a and IgG2b induction (Figure 5E and 5F). The decrease in IgG1 was less pronounced but did show a 3- to 5-fold decrease. In a separate experiment, CD40L blockade was carried out for only a short period, from the time of B-cell transfer to 6 d after. Twenty-two days following the last treatment (28 d after B-cell transfer), IgG2a (Figure 5G) and IgG2b (Figure 5H) levels were still reduced in the treated groups, even though B cells were still detected in the hosts. These experiments suggest that B-cell help in this model is delivered by a combination of soluble and surface molecules and is not a consequence of a single, tolerance resistant factor or skewed differentiation of the adapted T cells.

### Despite Help, Autoimmune Pathology Does Not Develop in a T Cell-Replete Host

**Figure 6:**
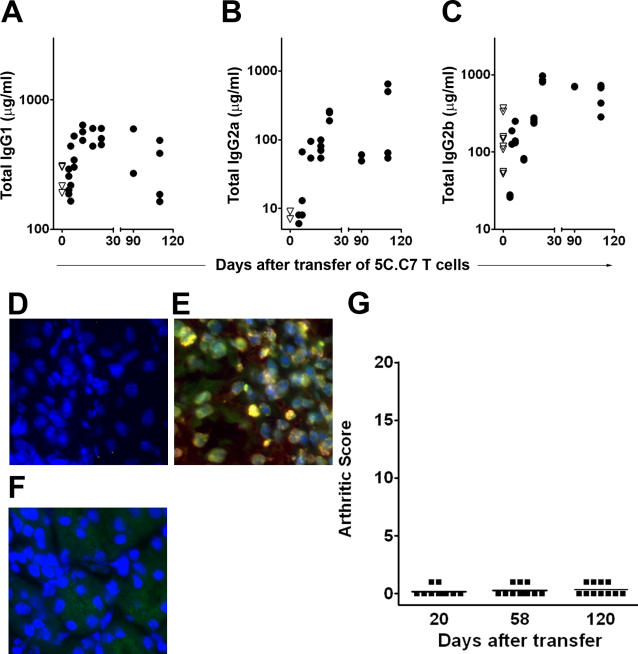
Autoantibodies and Arthritis Do Not Develop in the Presence of Polyclonal Endogenous T Cells (A–C) 3 million Ly5.1^+^,5C.C7 T cells were transferred into mPCC^+/+^ (T cell–replete) mice that had an intact repertoire of polyclonal T cells. Serum antibody titers were measured at various days after transfer. Total IgG1 (A), IgG2a (B), and IgG2b (C) levels are shown (filled circles). Inverted open triangles represent titers before T cell transfer. Data are pooled from two separate experiments with two to five mice per time point. Similar results were obtained in two other experiments. (D–F) Serum from T cell–replete (F) or T cell–deficient (E) PCC transgenic mice were used to stain tester B10.A,Rag2^−/−^ kidney sections at 1:100 dilution. Staining with control serum from a normal B10.A mouse is shown in (D). (G) Development of arthritis scored in the T-replete mPCC transgenic, 5C.C7 transfer recipients.

The experiments so far clearly demonstrate that in a carefully constructed model system where adaptive tolerance is the only potential mechanism of tolerance to a peripheral self-antigen, T cell effector functions sufficient to mediate autoimmune pathology are still evident. We then asked if this pathology would still be initiated, if other mechanisms of immunoregulation are restored to the model system. This was tested by transferring congenic 5C.C7,Ly5.1^+^ T cells to a B10.A,mPCC (Ly5.2^+^) transgenic mouse that retained an intact repertoire of endogenous T cells (CD3ɛ^+/+^). Serum from these transfer recipients also showed a consistent increase in IgG1 (Figure 6A), IgG2a (Figure 6B), and IgG2b (Figure 6C) levels. The titers of these antibodies, however, were approximately 5- to 10-fold lower than that observed in a T cell–deficient host (compare to Figure 3). Furthermore, the T cell–replete transfer recipients did not develop detectable titers of anticellular antibodies (Figure 6F) compared to the T cell–deficient host (Figure 6E). Consistent with these lowered titers and absence of anticellular antibodies, the transfer recipients that had an intact cohort of endogenous T cells did not develop detectable arthritic pathology even 120 d after T cell transfer (Figure 6G).

### 5C.C7 T Cells Are Anergic in a T Cell–Replete Host, but Do Not Undergo Differentiation toward Effector Function

**Figure 7:**
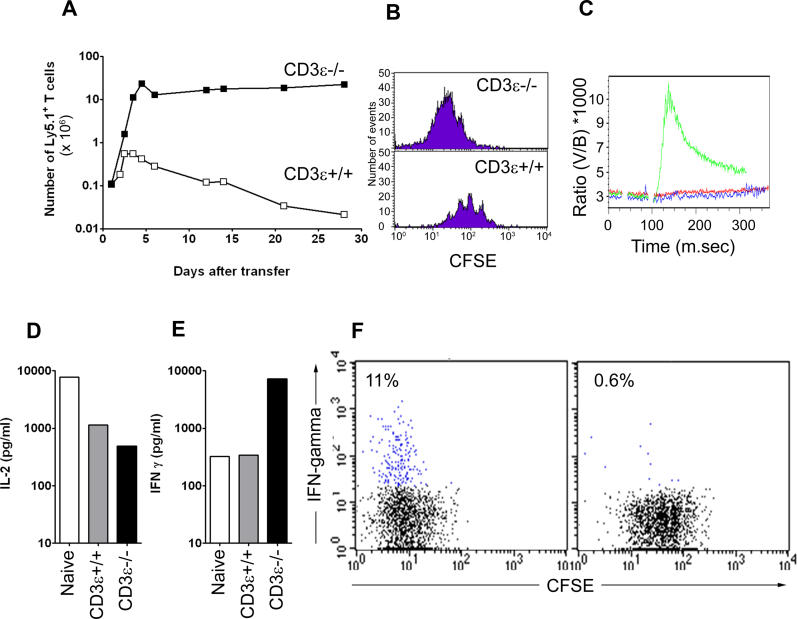
Poor Differentiation and Survival Are Superimposed on Adaptive Tolerance in the Presence of Endogenous T Cells (A) The total recovery of Ly5.1^+^ 5C.C7 T cells from lymph nodes and spleen of recipient mice, various days after transfer into a T cell–replete (mPCC^+^,CD3ɛ^+^; open squares) host are compared with the recovery from a T cell–deficient (mPCC^+^,CD3ɛ^−/−^ ; filled squares) transfer recipient. (B) Carboxy fluorescein succinimidyl ester profiles of transferred Ly5.1^+^ 5C.C7 T cells, 3 d after transfer into a lymphopenic (top) or T cell–replete (bottom) PCC transgenic host. (C) Ly5.1^+^,5C.C7 T cells, recovered 9 d after transfer to a T cell–replete PCC transgenic (red line) or T cell–deficient PCC transgenic (blue line) host, were loaded with Indo-1 and stimulated as described in Materials and Methods. The influx of calcium, measured as the ratio of violet to blue fluorescence, is compared to that of naïve 5C.C7 T cells (green line). (D) IL-2 or (E) IFN-γ secretion from sorted Ly5.1^+^,5C.C7 T cells restimulated in vitro with 10 μM MCC peptide and irradiated syngeneic splenocytes for 48 h, 15 d after residence in a T cell–replete (gray bars) or T cell–deficient (black bars) PCC transgenic hosts. Naïve 5C.C7 T cells (white bars) were used for comparison. (F) Ex vivo IFNγ secretion by transferred T cells recovered 4 d after transfer to a T cell–replete (right) or T cell–deficient (left) PCC transgenic host. FACS plots are gated on CD4+, 7AAD-ve cells in the lymphocyte area.

Given the striking difference in pathology observed in the presence or absence of endogenous T cells, we further dissected the fate of 5C.C7 T cells in the T cell–replete host. The transferred cells engrafted similarly in the T cell–replete host and initiated clonal expansion (Figure 7A, open squares). However, this expansion lasted only for 3 d and resulted in just a 5-fold increase in cell number. This was a consequence of a failure to sustain cell divisions, as evidenced by the carboxy fluorescein succinimidyl ester profiles (Figure 7B). Furthermore, the expanded T cells underwent a slow deletion process in the T cell–replete host, starting at day 4 and resulting subsequently in a approximately 3.5% per day loss of the expanded cells. This resulted in the reduction of the T cell numbers to below detection levels by approximately 2 mo. This disappearance phase was in sharp contrast to the fate of the autoreactive CD4^+^ T cells in the CD3ɛ^−/−^ (T cell–deficient) PCC transgenic host (Figure 7A, solid squares), which persisted for several months.

We then asked if an adaptively tolerant form of anergy was in fact induced in the 5C.C7 T cells in the T cell–replete host, by measuring IL-2 production and TCR proximal signal transduction. Sorted 5C.C7 T cells 15 d after adoptive transfer into a B10.A,mPCC+ host showed an 85% downregulation of IL-2 secretion (Figure 7D, gray bar, compared to naïve T cells in white bar) similar to the 93% downregulation in a CD3ɛ^−/−^ host. Over five separate experiments, 5C.C7 T cells in a T cell–replete host showed an 85 ± 3.5% decrease in IL-2 production relative to naïve 5C.C7 T cells, while in the lymphopenic host, this was 91 ± 7%. The CD3 proximal signaling machinery of the cells in either host was also tested by monitoring calcium influx in response to cross-linking with anti-CD3 ex vivo. The fluxes elicited by T cells from a T cell–replete host (Figure 7C, red trace) were equally dampened in comparison to the fluxes from those in a T cell–deficient host (Figure 7C, blue trace). Both were impaired in comparison to naïve T cells (Figure 7C, green line) as previously reported [27]. Therefore, the adaptive tolerance induced in the 5C.C7 cells is T cell–intrinsic and independent of the presence of an endogenous host repertoire of other T cells.

Finally, we found that the adaptively tolerant 5C.C7 T cells recovered from a T cell–replete host showed no significant differentiation towards effector cytokine production. Upon restimulation in vitro, adaptively tolerant T cells from the CD3ɛ^+/+^ host (Figure 7E, gray bar) made the same small amount of IFNγ as undifferentiated naïve T cell cultures (Figure 7E, white bar). T cells from a CD3ɛ^−/−^ host showed considerable differentiation to make this cytokine (Figure 7E, black bar), although this was, as reported earlier, still greatly impaired relative to a memory 5C.C7 T cell [7]. We further confirmed that autoreactive T cells fail to differentiate and display effector functions in a T cell–replete host by assaying for IFNγ secretion ex vivo, using a cytokine secretion assay (Figure 7F). About 11% of the adaptively tolerant 5C.C7 T cells were secreting this cytokine 4 d after transfer to a CD3ɛ^−/−^ host (Figure 7F, left), whereas only 0.6% of the cells could be scored positive in a CD3ɛ^+/+^ host. Similar to the lymphopenic host, 5C.C7 T cells in the T cell–replete host did not express detectable IL-4 or *foxp3* or upregulate CD25, suggesting that they had not differentiated into a Th2 or T-reg lineage (data not shown). Therefore, although adaptive tolerance was primarily induced in every context, a superimposing influence of the endogenous T cells is critical to reduce the expansion, effector differentiation and pathology stemming from autoreactive CD4^+^ T cells.

## Discussion

Anergy, deletion, and regulation have been proposed as three mechanisms that the adaptive immune system uses to tolerize CD4^+^ T cells reactive to peripheral self-antigens [4,30,31]. The relative significance of each of these processes to systemic tolerance and their synergism in vivo has been difficult to dissect, despite the existence of many model systems. It is formally possible that all of these mechanisms are in fact inseparable elements of a unified tolerance process. For example, the facts that regulatory cells display evidence of anergy and that anergic cells have been shown to regulate could be interpreted as evidence of similar biochemical machinery operating in both conditions. The conditions of assay or the quantitative gradation of the biochemical process may reveal anergy, regulation, or both. Therefore, it is critical to begin to separately quiz the roles of individual processes in peripheral tolerance and to decipher the network of interactions between these processes in vivo. This report represents a comprehensive examination of the role of in vivo anergy (adaptive tolerance) in the maintenance of peripheral tolerance. The adoptive transfer strategy we have used mimics the stimulation of a naïve T cell with a peripheral self-antigen or a chronic pathogen, albeit a systemic one. In most model systems, these responses are influenced by various noncell autonomous factors such as regulatory T cells, Th2 or Th3 cells, NK T cells, γδ T cells, etc. The absence of these other endogenous T cells in the recipient we have used (CD3ɛ^−/−^ host) limits the regulatory mechanisms available to those intrinsic to the responding T cell population. We also eliminated the induction of trans-regulatory phenotypes within the population we have introduced, as has been found in other models [19,32]. Thus, our data clearly demonstrate that while tuning effectively constrains clonal expansion and quantitatively buffers cytokine synthesis, it is by itself unable to prevent the differentiation and survival of autoreactive T cells, two parameters that could very well be linked [33]. These parameters must be constrained by other non–cell-intrinsic regulators, so as to completely eliminate pathology.

The conclusions of this study are supported by earlier arguments that anergic T cells can help B cells generate a Th1 (IgG2a) class of response [10]. Those data, however, were consistent with the “overcoming” of anergy by a stronger antigenic challenge. In contrast, the helper activity in our model is continual and does not require an antigenic challenge exceeding the level of ambient presentation. Furthermore, the differentiation in this system does not skew the responses toward any particular class of antibody production. Antibodies typically associated with Th1 (IgG2a) as well as Th2 (IgG1 and IgE) helper function were all upregulated. The increased Ig levels did not just reflect specific antibody production against the cognate antigen (PCC, which was absent in most mice) but was of diffuse polyclonal reactivity (Figure 3). This is not surprising, since the expression of PCC under the control of the MHC-I promoter and Ig enhancer should result in antigen expression in all of the B cells [7,17]. Endogenous membrane bound antigens have been shown to gain access to the class II pathway during membrane recycling or by autophagy [34–36]. This should allow all B cells in this model system to express PCC peptide–MHC complexes and thus interact with the 5C.C7 T cells independent of the B cell's own receptor specificity. This in turn would generate an efficient mechanism for providing T cell help to any B cell whose Ig receptors also engaged their cognate antigen, including abundant self-antigens that might normally rely on the absence of T cell help to prevent autoantibody generation. In fact, among the antibodies generated in our system, some were reactive against DNA, histones, and GPI molecules implicated in B-cell–mediated autoimmune disorders like arthritis or lupus in other mouse model systems [37]. The transfer recipients in our model progress toward a mild form of arthritis (Figure 3). This disease did not develop in the absence of B cells (Rag2^−/−^ PCC^+^ transfer recipients) at least up to 110 d after T cell transfer (data not shown). Although the simplest interpretation of these data would be that T cell–dependent antibody production is the etiologic agent in the disease process, we have not excluded the possibility that the presence of B cells in the system merely enhanced a pathologic T cell response in this model. In preliminary experiments transfer of serum from arthritic mPCC,CD3ɛ^−/−^ mice to mPCC,RAG2^−/−^ mice did not result in the induction of arthritis in the recipients (unpublished data). A further complication is that in the mPCC,RAG2^−/−^ recipients the plateau levels of 5C.C7 T cells are approximately 4-fold lower than that in the mPCC,CD3ɛ^−/−^ host. Nonetheless, the disease process is clearly T cell initiated, whether through helping B cells or triggering other inflammatory cascades in the innate immune system [37,38]. In either case, it reflects continued effector responses from the peripherally “tuned” T cells.

Importantly, the B-cell–deficient system was useful to show that continued help is available to B cells even after the T cells have adapted (Figure 4). Interestingly, the arthritic pathology in this case was delayed by over a month and even then significantly reduced compared to that in the CD3ɛ^−/−^ host (Figures 2F and 4I). Again, although it is arguable that the delay in pathology represents a consequence of anergy induction, we cannot rule out the possibility that this was due to the 4-fold lower number of anergic T cells or the absence of continued B-cell generation in the same host resulting in a reduced “repertoire” of B cells. The ability of the B cells to receive help is still very surprising, given our earlier report that dendritic cells isolated from this antigen transgenic mouse present higher levels of antigen than resting B cells in an in vitro T cell proliferation assay [7]. One might have predicted that a quantitative adaptation to the level of antigen presentation by dendritic cells should have abrogated the T cell's ability to productively sense any antigen on B cells. Or observations therefore argue against quantitative receptor calibration being sufficient as a tolerance mechanism, at least for a high-affinity receptor–ligand pair in the periphery.

The ability of a broad repertoire of B cells to present antigen makes our system comparable to models of persistent viral infection, like the LCMV model. Interestingly, such infections also induce a polyclonal hypergammaglobulinemia that is strictly dependent on viral antigen-specific T cell help [15]. Virus-specific helper T cells during chronic infections also enter an anergic state within 2 wk and, in seeming progression of the adaptation, subsequently lose the ability to prime de novo B-cell responses [15,39]. This difference from our results might stem from intrinsic differences in the model systems. These include the generation of antibody escape variants due to viral replication, the presence of virus derived TLR ligands, and the possibility that constant fluxes in antigen dose may recruit mechanisms other than tuning to regulate immunopathology [40–42]. Furthermore, the use of mice with an intact repertoire of T cells in the LCMV infections allows for non–cell-autonomous regulatory mechanisms to be superimposed on the adaptive tolerance process (as we discussed earlier). Nonetheless, it is interesting to note that even during persistent viral infections, CD4^+^ T cells are required for effective control of the virus [43,44]. In cases where chronic viral infections have been shown to abrogate effector functions in CD8^+^ T cells, the viremia is still constrained progressively and the virus distribution localized to restricted tissues, arguing that biological activity in the CD4^+^ or CD8^+^ cells is being maintained despite the persistent infection [13]. These experiments, however, need to be reexamined in the absence of thymic output and trans-regulatory cells, using adoptive transfer systems similar to the one we have used.

The restoration of an intact polyclonal T cell compartment in our experimental model (Figures 6 and 7) was sufficient to constrain the autoimmune pathology that we observed. This allowed us to investigate the cellular paradigm that needs to superimpose on adaptive tolerance, in order to achieve “complete” tolerance. Interestingly, although adaptive tolerance (decreased TCR signaling and IL-2 production) was induced to equal extents without regard to the presence of other T cells, the subsequent elimination of autoreactive T cells only happens in the presence of other endogenous T cells. These data demonstrate that while anergy is chosen as a primary, cell-intrinsic fate, following tolerogenic antigen encounter in the periphery, the subsequent “deletion” is unlikely to be cell autonomous. The nature of the T-T interaction that results in the slow decay of the anergic T cell population is under investigation. Preliminary experiments using anti-CD25 and anti–IL-2 treatment to deplete CD25^+^ T cells in the T cell–replete host suggest that such cells are unlikely to be sufficient to mediate this process. Alternatively, we favor a trophic competition process similar to the one that has been described for B cells [45]. The process is likely to be more than a mere competition for “space,” since preliminary experiments suggest that simply filling up the animal with a second TCR transgenic CD4+ population is insufficient to eliminate the adapted 5C.C7 T cell. However, the recent demonstration that clonal competition between T cells can affect proliferation and survival in vivo may be relevant to the control of autoimmune pathology in this regard [46,47]. A similar paradigm may also operate in the case of CD8+ T cells that are rendered tolerant to self-antigen [48].

Unlike thymic negative selection or activation-induced cell death, the “deletional” component of peripheral T cell tolerance seems to be a relatively slow process. While the reduction of T cell numbers as a result of it is indeed quite striking, it still does not prevent the persistence of a large number of autoreactive T cells in the animal for up to 2 mo. Thus, even in the T cell–replete host, the adaptive tolerance component must play a critical role in constraining the renewed recruitment and expansion of the high affinity autoreactive T cells. In addition, the inability of these CD4^+^ T cells to fully turn on autoreactive B cells likely stems from the profound blockade in the differentiation of these cells in the T cell–replete host. T cell differentiation to acquire effector functions have previously been correlated with the extent of cell division [49]. The limited initial expansion of the T cells could therefore lead to poor expression of effector molecules (including IL-4, IFNγ, IL-21, and CD40L) and handicap the ability of adapted T cells to help B cells. Such an impairment of effector differentiation (and migration) of pathogenic T cells can also be achieved by the action of trans-regulatory T cells as observed in other model systems [50,51].

Multiple theories have debated the merit of singular mechanisms that maintain peripheral tolerance (reviewed in [52]). Models of T cell tuning have argued that a quantitative dampening of the T cell's sensitivity to ambient antigen should be sufficient to prevent pathology [53,54]. The maintenance of such cellular tuning requires that the cell be constantly aware of the persistence of antigen. The prevention of pathology, on the other hand, requires the cell to avoid effector responses despite the constant interaction of the receptor with a strong endogenous agonist. The success of tuning as a tolerance mechanism therefore depends on the ability of the T cell to walk this biochemical tightrope efficiently. Our data would argue that over time, in the absence of additional mechanisms of peripheral tolerance, enough helper activity is able to break through, so as to mediate visible systemic pathology. It is quite likely that this inadequacy is not unique to the mechanism of adaptive tolerance, because it has been argued that other mechanisms of immune regulation, e.g., T-regs, also cannot control autoimmunity in isolation [55]. Nonetheless, quantitative dampening of TCR responsiveness (and the kinetic delay in the onset of pathology that it might afford [Figure 4F]) could in fact be quite critical in vivo, allowing additional regulatory processes to effectively collaborate and enforce complete peripheral tolerance [56].

## Materials and Methods

### Mice and cells.

Mice used in this study were obtained through the NIAID contract at Taconic Farms (Germantown, New York, United States), an AAALAC accredited facility. All the mice used in these experiments were bred on a B10.A (H-2^a^) background and were Ly5.2^+/+^ unless otherwise noted. The 5C.C7,Rag2^−/−^; mPCC transgenic; CD3ɛ^−/−^; and mPCC,CD3ɛ^−/−^ strains have been described [6,7]. mPCC,CD3ɛ^−/−^ mice were crossed twice to Rag2^−/−^ animals selecting for Rag2^−/−^ homozygotes, eliminating the CD3ɛ-null allele and intercrossed, to derive the B10.A,mPCC,Rag2^−/−^ strain. The 5C.C7,Rag2^−/−^,Ly5.1^+/+^ strain was derived by crossing 5C.C7,Rag2^−/−^ mice to B10.A,Ly5.1^+/+^. The 5C.C7,Rag2^−/−^,IL-4^−/−^ strain was a gift from J. Zhu and W. E. Paul (NIAID, NIH, Bethesda, Maryland, United States). The IFNγ-deficient allele was backcrossed onto the B10.A,5C.C7,Rag2^−/−^ background three times from a B6,IFN-γ^−/−^ (N12) strain. The B6,IL21R^−/−^ mouse was obtained from R. Spolski and W. Leonard (NIAID, NIH), crossed to the B10.A background four times, and rendered homozygous for both the mPCC transgene and the CD3ɛ^−/−^ allele.

For adoptive transfer experiments involving T cells from 5C.C7 TCR transgenic mice, lymph node suspensions containing greater than 90% CD4+ T cells (1 to 3 million per recipient) were injected without further purification into recipients by the suborbital route. B cells were purified from the spleens of either B10.A,mPCC,CD3ɛ^−/−^ or B10.A,CD3ɛ^−/−^ mice using the mouse B-cell purification kit (Miltenyi Biotech, Auburn, California, United States) or sorted to greater than 98% purity after labeling with anti-B220 PE (BD Biosciences, San Diego, California, United States). Twenty-five million purified B cells were transferred in the experiments shown, but injections of up to 50 million B cells gave similar results.

In vivo blockade of CD40L was achieved by injecting 100 μg/d mAb MR1 and compared with a control hamster IgG (anti-TNP), both obtained as no azide/low endotoxin grade from BD Biosciences.

### In vitro T cell cultures and functional assays.

Transferred T cells stained with anti-CD45.1 PE and anti-CD4 APC (BD Biosciences) were sorted to greater than 99% purity using a BD Vantage or BD Aria sorter at the NIAID flow cytometry facility. Ten thousand sorted T cells were cocultured with 2 to 5 × 10^5^ irradiated B10.A,CD3ɛ^−/−^ splenocytes and titrations of Moth Cytochrome C (MCC) 88–103 peptide (American Peptide Co., Sunnyvale, California, United States). Culture supernatants taken 48 h later were assayed by ELISA for IL-2 and IFNγ secretion as per manufacturer's instructions (R&D Systems, Minneapolis, Minnesota, United States). Ex vivo analysis of cytokine secretion was carried out using the IFNγ secretion assay (Miltenyi Biotec) as previously described for IL-2 [57]. Analysis was restricted to viable cells based on the exclusion of the vital dye 7-AAD (BD Biosciences). Intracellular *foxp3* expression was measured using the FKJ-16s staining kit (eBioscience, San Diego, California, United States) per the manufacturer's instructions. Calcium responses in T cells were measured by loading cells with 5 μg/ml Indo-1 (Molecular Probes, Eugene, Oregon, United States) at 37 °C before stimulation. Loaded cells were washed in cold HBSS containing 1 mM CaCl_2_ and 1 mM MgCl_2_, before labeling with Ly5.1-PE and CD4-APC at 4 °C. The washed cells were briefly warmed to 37 °C before analyzing on a BD LSR II cytometer. After 30 s, prewarmed biotinylated anti-CD3 was added to a final concentration of 10 μg/ml, and 2 min later, a warm solution of streptavidin (5 μg/ml final) was added and the data were collected for a further 400 s. Collected data were analyzed using the FlowJo software (Treestar, Ashland, Oregon, United States).

### Antibody measurements.

Serum samples were assayed for various antibodies using sandwich ELISAs. For determination of total globulin levels, anti-mouse kappa or anti-mouse lambda (Caltag, Burlingame, California, United States) was coated on NUNC Maxisorp plates (Nalge Nunc, Rochester, New York, United States) at 1 μg/ml concentrations in carbonate buffer (pH 9.0). Binding was blocked with 0.25% BSA in Tris-buffered saline + 0.1% Tween-20 (TBS-Tween). Serum dilutions made in TBS-Tween were incubated with the plates and the bound antibodies detected with isotype-specific antisera coupled to HRP (Caltag). The concentrations of each isotype were calculated from a standard curve using purified Ig isotypes (CaltagA). Anti-DNA antibodies were assayed similarly, after coating with calf thymus DNA (10 μg/ml) instead of anti-lambda/kappa.

### Western blots.

Purified proteins were loaded at 1 μg/well and separated on a 5% to 12.5% PAGE gel (Bio-Rad, Hercules, California, United States). Separated proteins were transferred onto nitrocellulose and bound with serum samples diluted 1:1,000 in TBS-Tween. Bound antisera were detected with anti-mouse Ig labeled with HRP (Bio-Rad).

### Immunohistochemistry.

Formalin-fixed and decalcified limb samples were mounted on paraffin blocks and sectioned to 15-μm thickness before processing for hematoxylin and eosin staining. For ANA analysis, healthy B10.A,Rag2^−/−^ kidneys were cryosectioned onto silanized slides by Histoserv Inc. (Gaithersburg, Maryland, United States). Each slide had duplicate sections of such kidneys and were fixed in Histochoice solution (Amresco, Solon, Ohio, United States) for 2 to 3 min. Nonspecific binding was blocked with 1% BSA and donkey serum, followed by incubations with serum samples diluted 1:3,000. Bound antibody was detected with Fab fragments of donkey anti-mouse IgM and donkey anti-mouse IgG, labeled with Cy3 and FITC, respectively (Jackson ImmunoResearch, West Grove, Pennsylvania, United States). Stained sections were scored as positive on a Leica DMRX fluorescent microscope if both duplicate sections on the slide were stained positive. Images were collected using a CCD camera and Slidebook software (Intelligent Imaging, Petaluma, California, United States). The range of colors observed depended on the relative amounts of IgM (red) and IgG (green) antibodies in the tested serum that bind self-antigens in the kidney sections. Overlap of red and green signals at high intensity resulted in the yellow colors on the composite images.

### Arthritic score.

An arbitrary arthritic score was developed to quantitate the extent of disease progression in the mice. Swelling in individual digits was assigned a score of 1 (per digit) with an additional score of 1 added if the digits were inextensibly curled up. Thus each leg could get a maximum theoretical score of 10 based on the health of the digits (counting the polex as the fifth toe in the front legs). Swelling of the feet and lower legs were assigned scores of one each increasing the maximum score per leg to 12. In addition, the inability to extend the elbow or knee joints fully was given a score of one per leg, while the failure to extend this joint beyond a approximately 45-degree angle was scored as 2. As a result, the maximum score per animal in this system was 56 (14 × 4). The shoulders and thighs were not scored in these mice.

### X-ray imaging.

Mice were killed and taped to film before being exposed to 30-kV x-rays for 55 seconds at the NICHD faxitron facility (Bethesda, Maryland, United States).

### Statistical analysis.

All statistical analyses were done using the unpaired, two-tailed Student's *t*-test and Prism 4 software (GraphPAD, San Diego, California, United States).

## Abbreviations

GPI: glucose-6-phosphate isomerase
IFN: interferon
IL: interluekin
LCMV: Lymphocytic choriomeningitis virus
PCC: pigeon cytochrome C
TCR: T cell receptor
